# The Cost of Nursing per Bed

**Published:** 1909-07-03

**Authors:** 


### Nursing Administration.

### THE COST OF NURSING PER BED.

In a preceding article the cost of each nurse to the hospital, less the collective items included in the nursing homes and shared by all alike, was examined, the figures being taken, together with those in this article, from *Burdett's Hospitals and Charities*, 1909. The tables showed that while very little difference is to be found in the style of living at these various establishments, the bill of fare for the nurses presenting practically the same features in each, the cost was at least £18 more per year a head, if uniform be reckoned, in the London Hospital than in Guy's, the one which headed the list for economy. In provincial hospitals it cost £7.2 a year more per head at the Leeds General Infirmary for the maintenance of the nurse than at the Radcliffe, Oxford. In Scotland a nurse cost £9.8 more a year at the Glasgow Royal Infirmary than at the Dundee Royal, and £7.7 more than at the Edinburgh Royal Infirmary. There are 251 nurses at Guy's Hospital. If the cost of each nurse were increased by £18, the additional cost to the hospital would be £4,518. There are 441 nurses at the London Hospital. If the cost of each nurse were reduced by £18, the saving effected would be £7,938. If the Royal Edinburgh Infirmary spent as much over each nurse as the Glasgow Royal, it would make a difference of over £2,094 in the yearly cost. If the Glasgow Royal could reduce the cost of each nurse to the level of the Dundee Royal, it would save £1,666 a year.

The cost of the nursing in any hospital per bed depends mainly, though not exclusively, on the average number of nurses employed in proportion to the patients, a proportion generally estimated by excluding such nurses as are engaged in out-patient work or in other departments outside the wards, and by dividing the number of beds among the remainder. Nurses required to enable the staff to take leave and holidays are included in these totals, experience showing that skill in organisation makes a wide difference in the numbers required for this purpose. It is to be observed that decimals are of considerable importance in this mode of reckoning, it having been calculated by the author of "The Matron's Duties and Responsibilities"* that "the difference between 2 beds and 2.1 beds apiece is almost exactly a difference of two nurses on every 100 beds."

The curious thing in the following table is that whereas in Scotland there is but just over 30s. between the highest and the lowest average, and only £3 12s. difference in the provinces, yet in London the variation in cost is £11.1, extending from £18.2 to £29.3, the latter figure not including uniforms. It would cost Guy's £5,716.5 to raise the expenditure on the nursing per bed in proportion to that at the London. The London Hospital would save £8,791.2 per year if it could reduce the cost of the nursing per bed to the Guy's average. It does not by any means follow that the cost of nursing at one hospital can be made to approximate to that at another. But it is remarkable that such wide variations are seen to occur in the same class of hospital and in the same town.

* London: The Scientific Press, 28 & 29 Southampton Street, Strand.

Table A.—HOSPITALS WITH MEDICAL SCHOOLS.

| | Daily Average of Occupied Beds | Total Nursing Staff | Average No. of Occupied Beds to Each Member of Day and Night Staff | Cost of Nursing per Bed £ |
|---|---|---|---|---|
| *London—* | | | | |
| Guy's ... ... ... | 515 | 251 | 2.3 | 18.2 |
| University College ... | 244 | 103 | 2.7 | 17.5* |
| Royal Free ... ... | 138 | 62 | 2.3 | 19.8 |
| St. Mary's | 257 | 112 | 2.6 | 20.9 |
| King's College ... ... | 189 | 87 | 2.3 | 21.0 |
| St. Thomas's ... ... | 480 | 192 | 2.6 | 21.2 |
| Middlesex ... ... | 270 | 117 | 2.5 | 22.6 |
| St. George's ... ... | 319 | 147 | 2.3 | 24.5 |
| St. Bartholomew's ... | 575 | 288 | 2.2 | 25.4 |
| London ... ... ... | 792 | 441 | 2.0 | 29.3* |
| *Provincial—* | | | | |
| Leeds General Inf. ... | 368 | 95 | 4.0 | 11.0 |
| Oxford Radcliffe ... | 116 | 41 | 3.4 | 12.6 |
| Cambridge Addenbrookes | 107 | 37 | 3.2 | 13.6 |
| Birmingham General ... | 305 | 106 | 3.2 | 13.7 |
| Birmingham (Queen's) | 121 | 44 | 3.1 | 14.6 |
| *Scotch—* | | | | |
| Dundee Royal Inf. ... | 276 | 94 | 3.3 | 12.1 |
| Edinburgh Royal Inf.... | 832 | 272 | 3.2 | 12.3 |
| Glasgow Western Inf. | 544 | 176 | 3.3 | 12.7 |
| Glasgow Royal Inf. ... | 593 | 170 | 3.8 | 13.0 |
| Aberdeen Royal Inf. ... | 226 | 81 | 3.0 | 13.7 |
| *Irish—* | | | | |
| Belfast Royal Victoria | 223 | 77 | 3.2 | 13.7 |

* Minus uniforms.

The variations in cost displayed in Table B are even more striking, the cost of the nursing per bed ranging from £12.7 at the Seamen's Hospital to as much as £31.4 at the Miller, where, however, the enormous out-patient department in proportion to the small number of beds must be allowed for. In the provinces the range of variation in the medium-sized General Hospitals is £11.2, from £9.6 at the Kent and Canterbury to £20.8 at the Chester General.

Table B.—GENERAL HOSPITALS WITHOUT MEDICAL SCHOOLS.

| | Daily Average of Occupied Beds | Total Nursing Staff | Average No. of Occupied Beds to Each Member of Day and Night Staff | Cost of Nursing per Bed £ |
|---|---|---|---|---|
| *London—* | | | | |
| Seamen's ... ... | 247 | 64 | 4.3 | 12.7 |
| Great Northern Central | 147 | 54 | 3.1 | 16.0 |
| Bolingbroke ... ... | 29.5 | 18 | 1.9 | 20.6 |
| Poplar ... ... ... | 78 | 36 | 2.4 | 23.8 |
| Miller General ... | 22.1 | 13 | 3.1 | 31.4 |
| *Provincial—* | | | | |
| Kent and Canterbury | 77 | 23 | 3.6 | 9.6 |
| Northampton General | 148 | 44 | 3.7 | 10.9 |
| East Suffolk & Ipswich | 105 | 30 | 3.8 | 11.0 |
| Stoke-on-Trent Inf. ... | 165 | 50 | 3.5 | 11.6 |
| Worcester General ... | 114 | 33 | 4.3 | 11.7 |
| Bradford Royal Inf.... | 181 | 61 | 3.5 | 12.5 |
| Wolverhampton & Staff. | 150 | 52 | 3.2 | 12.6 |
| Cardiff Infirmary ... | 168 | 57 | 3.3 | 13.3 |
| York County ... ... | 101 | 41 | 2.8 | 14.2 |
| Royal Devon & Exeter | 155 | 52 | 3.4 | 15.0 |
| Norfolk and Norwich | 166 | 54 | 3.6 | 15.6 |
| Royal Berkshire ... | 145 | 51 | 3.2 | 16.6 |
| Leicester Infirmary ... | 176 | 64 | 3.1 | 17.3 |
| Taunton and Somerset | 90 | 43 | 2.3 | 19.9 |
| Chester General ... | 93 | 44 | 2.4 | 20.8 |
| *Scotch—* | | | | |
| Ayr County ... ... | 48 | 18 | 3.0 | 14.0 |
| Dumfries and Galloway | 73 | 25 | 3.3 | 14.8 |

